# A Preliminary Study on the Effects of Black Cohosh Preparations on Bone Metabolism of Rat Models With GnRH-a-Induced Peri-Menopausal Symptoms

**DOI:** 10.3389/fendo.2022.854345

**Published:** 2022-06-09

**Authors:** Zhenyue Qin, Zhiyong Dong, Junling Liu, Ahong Zhong, Mingyue Bao, Huihui Wang, Hongxia Yu, Shoufeng Zhang, Wendi Zhang, Li Shen, Jie Wu, Jiming Chen

**Affiliations:** ^1^ Department of Obstetrics and Gynecology, The Affiliated Changzhou No.2 People’s Hospital of Nanjing Medical University, Changzhou, China; ^2^ State Key Laboratory of Reproductive Medicine, Nanjing Medical University；Department of Obstetrics and Gynecology, The First Affiliated Hospital of Nanjing Medical University, Nanjing, China

**Keywords:** black cohosh preparations, GnRH-a injection, peri-menopausal symptom rat models, bone metabolism, preliminary study

## Abstract

**Background:**

Endometriosis (EMS) is a relapsing and estrogen-dependent disease. For endometriosis such as deep endometriosis and ovarian endometrioid cysts, surgery is the most effective treatment. Long-term follow-up showed that the recurrence rate of endometriosis after surgical treatment was high, so postoperative drugs were needed to reduce recurrence, and Gonadotropin-releasing hormone agonists (GnRH-a) were the most commonly used drug for postoperative management.GnRH-a may reduce the post-treatment endometriosis relapses by lowering the hormone levels in the body. However, the use of GnRH-a can give rise to perimenopausal symptoms, especially osteoporosis, bone loss, and bone pain, for which reason GnRH-a use is often limited. The add-back therapy is often used to alleviate the untoward effects caused by GnRH-a. However, long-term use of hormone drugs may lead to EMS recurrence, thrombosis, and breast cancer. Therefore, a safer and more effective drug is urgently needed to alleviate the untoward effects caused by GnRH-a. In recent years, scholars at home and abroad have found that isopropanolic Cimicifuga racemosa extract (ICR), as a plant extract, can better relieve the symptoms of perimenopausal women. At the same time, some studies have initially confirmed that black cohosh preparations can relieve the perimenopausal symptoms caused by GnRH-a treatment in EMS patients.

**Objective:**

To investigate the effect of black cohosh preparations on the bone metabolism of rat models with GnRH-a-induced perimenopausal symptoms.

**Methods:**

The rat models of perimenopausal symptoms were established by GnRH-a injection. and normal saline (NS injection) was used as the control. According to the modeling method and drug intervention, the rats were randomly divided into four groups: GnRH-a injection + saline intervention group (GnRH-a + NS), saline injection control + saline intervention group (NS + NS), GnRH-a injection + estradiol intervention group (GnRH-a + E2), and GnRH-a injection + black cohosh preparation intervention group (GnRH-a + ICR). The rat models were identified with the vaginal smear method, and then the corresponding drug intervention was administrated for 28 days. After the intervention, the rats were sacrificed. The rats’ bone mineral density (BMD) of the distal femur was detected by a dual-energy X-ray bone density scanner. Rat tibia bone tissues were decalcified and made into slices. The pathological and morphological changes of rat tibial bones in each group were observed through HE staining. Histomorphometry parameters of rat tibial bones in each group, such as trabecular bone volume (TBV), trabecular thickness (TbTh), trabecular number (TbN), and trabecular spacing (TbSp), were detected and analyzed by using an automatic image analysis system.

**Results:**

(1) The BMD level of the distal femur in the GnRH-a + NS group was significantly lower than the NS + NS, GnRH-a + E2, and GnRH-a + ICR groups (*P*<0.01), the BMD levels in GnRH-a + E2 and GnRH-a + ICR groups were slightly lower than the NS + NS group, but there was no significant difference among the three groups (*P*>0.05). (2) The pathological changes of the tibia bones under the microscope in different groups were as follows: The tibia bone trabecular structure was normal in the NS + NS group, without trabecular thinning or fracture, and the arch structure was normal. In the GnRH-a + NS group, some trabecular structures tapered, the arch structure disappeared, but no obvious bone fracture was observed in the trabecula. In the GnRH-a + E2 and GnRH-a + ICR groups, the trabecular structures were normal, without trabecular bone thinning or fracture, and the arch structures were normal. (3) The TBV level of the GnRH-a + INS group was significantly lower than that of the NS + NS, GnRH-a + E2 and GnRH-a + ICR groups (*P*<0.01, *P*<0.05, *P*<0.01), while there was no significant difference among NS + NS, GnRH-a + E2 and GnRH-a + ICR groups (*P*>0.05). (4) The TbTh levels in the four groups had no significant difference (*P*>0.05). Compared with the NS + NS group, the TbTh levels in the GnRH-a + NS, GnRH-a + E2, and GnRH-a + ICR groups showed a descending tendency, while the TbTh levels in the GnRH-a + E2 and GnRH-a + ICR groups were slightly higher than that of the GnRH-a + NS group. However, such differences were not significant statistically (P>0.05). (5) Compared with the NS + NS group, the TbN levels in the GnRH-a + NS, GnRH-a + E2, and GnRH-a + ICR groups decreased remarkably (*P*<0.05). Compared with the GnRH-a + NS group, the TbN levels in the GnRH-a + E2 and GnRH-a + ICR groups showed a mild descending tendency, but such differences were not significant statistically (*P*>0.05). (6) The TbSp level of the GnRH-a + NS group was significantly higher than that of the NS + NS, GnRH-a + E2, and GnRH-a + ICR groups (*P*<0.01), while there was no significant difference among NS + NS, GnRH-a + E2 and GnRH-a + ICR groups (*P*>0.05).

**Conclusion:**

The GnRH-a injection could achieve the desired effect. GnRH-a injection may lead to the loss of bone mass in rats. Black cohosh preparations, like estrogen, may have a protective effect on bone mass loss caused by GnRH-a injection.

## Introduction

Endometriosis (EMS) is a common gynecological benign disease. About 10%-15% of women of childbearing age suffer from this disease (1), and the incidence has increased significantly in recent years (2). It also is one of the biggest challenges for gynecologists. For EMS treatment, laparoscopic surgery combined with drug therapy is currently the best combined solution. Adding gonadotropin-releasing hormone agonist (GnRH-a) after surgery can effectively prevent the disease recurrence (3). Main side effects after longtime use of GnRH-a are perimenopausal symptoms and osteoporosis caused by low estrogen. Long-term GnRH-a treatment will deteriorate bone loss, and some patients may suffer from severe bone pain symptoms. Some scholars have proposed to use the add-back therapy from the beginning of GnRH-a use (4). However, longtime use of hormones may increase the incidence of hormone-dependent diseases (5).

Black cohosh (cimicifugae racemosa, or CR) is a plant of the Ranunculaceae and is effective in relieving perimenopausal symptoms in postmenopausal women. However, most of the current studies focus on the treatment of healthy natural menopausal and perimenopausal women. According to related studies, the main physiological functions of black cohosh include (1): the effect on the reproductive endocrine system. Black cohosh does not increase the serum estrogen level or stimulate endometrial hyperplasia (6). Black cohosh extract had no estrogenic effect on the uterus and vagina. Its mechanism of action may be related to the fact that black cohosh contains both tissue-selective estrogenic agonists (estrogen-like effects in bones) and antagonists (anti-estrogenic effects in breast and endometrium) (7) (2). The role of black cohosh in the nervous system. Black cohosh can act on the 5-HT neurotransmitter system, which may be one of the reasons why black cohosh can relieve hot flashes and improve mood (3). The role of black cohosh in preventing osteoporosis. Black cohosh has an estrogen-like effect and can prevent and treat osteoporosis caused by a lack of estrogen. Some scholars have studied rats with artificially induced osteoporosis and found that black cohosh extract has a similar effect to that of raloxifene in treatment of osteoporosis (8). The study found that black cohosh extract can reduce the content of adrenocorticotropic hormone (ACTH) in the peripheral blood of ovariectomized rats and improve the osteoporosis of ovariectomized rats (9). Studies had also confirmed that the active ingredients of black cohosh can inhibit osteoclast-like cells (10) (4). The role in tumor inhibition. Compared with estrogen drugs, black cohosh has a significant advantage in that it not only won’t increase the risk of breast and endometrial cancer but also has a certain anticancer effect (11, 12) (5). The effect on the cardiovascular system. After treatment with isopropanol extract, the total cholesterol (TC) content did not change significantly, but the high-density cholesterol (HDL-C) increased significantly and the low-density cholesterol (LDL-C) decreased significantly. Therefore, it is inferred that the black cohosh extract can be used in cardiovascular disease treatment (13).

Black cohosh preparations can better relieve the perimenopausal symptoms of natural menopause, so we can infer that it can also effectively antagonize the perimenopausal symptoms of EMS patients treated with GnRH-a. However, the efficacy, safety, and mechanism of black cohosh preparations in antagonizing perimenopausal symptoms caused by GnRH-a, and whether black cohosh preparations can provide the same good bone protection as estrogen can are not clear, and need further discussions and studies. For this purpose, we have designed the following experiment to identify the effect of black cohosh preparations on bone metabolism in a rat model of GnRH-a-induced perimenopausal symptoms.

## Materials and Methods

### Materials

#### Experimental Animals

Twenty-four female Sprague-Dawley (SD) clean rats, aged 5 to 6 weeks and weighing (200 ± 20)g, were purchased from the Slac Laboratory Animal, with approval from the Experimental Animal Welfare Ethics Committee. The license number for experimental animals: SCXK(Hu) 2012-0002. Six rats/fed in polycarbonate cage with sufficient food and water. The rats were free to drink and eat during the experiment. The rats were fed with granular feed without soybean until the end of the experiment, so as to eliminate the influence of phytoestrogen on the result. The rats were kept at room temperature of (25 ± 1)°C, relative humidity of 50%-55%, and a circadian rhythm of 12 hours. After the rats got used to the feeding environment for one week, the experiment officially started. In this study, all experimental operations on the experimental animals were carried out by personnel with an experimental animal work certificate. All experimental operations complied with the Regulations on Experimental Animals of the People’s Republic of China promulgated by the State Scientific and Technological Commission of China and the Regulations on Laboratory Animals of Jiangsu Province.

#### Investigational Drugs and Reagents

(1) Black cohosh preparations (Schaper & Briimmer GmbH & Co. KG, Germany) (2). Estradiol valerate (Guangzhou Branch of Bayer Healthcare Co., Ltd.), trade name Progynova, 1 mg/tablet (3). Gonadotropin-releasing hormone agonist (GnRH-a): (Beaufour Ipsen France), 3.75 mg/injection (4). 0.9% saline for injection (0.9% saline, NS): (Zhejiang Jimin Pharmaceutical Factory) (5). 10% chloral hydrate:10g of chloral hydrate was dissolved in a proper amount of physiological saline, the volume was fixed to 100ml, and the mixture was fully stirred and mixed with an electromagnetic stirrer so that the 10% concentration required for injection was prepared.

#### Main Experimental Reagents

(1) Xylene (Chengdu Kelong Chemical Reagent Factory) (2); Absolute ethanol (Chengdu Kelong Chemical Reagent Factory) (3); Hematoxylin (SIGMA, batch number: 041M0014V) (4); Eosin (Shanghai Maikun Chemical Co., Ltd., batch number: 20120831) (5); Hydrochloric acid solution (Hangzhou Shuanglin Chemical Reagent Factory) (6); Neutral balsam (Shanghai Specimen Model Factory).

#### Main Instruments and Equipment

(1) Ultra-low temperature refrigerator (BS-812) (2); High-speed freezing centrifuge (Hunan Xiangyi Equipment, model TGL-16m) (3); Stereotaxic apparatus (Chengdu Taimeng Technology Co. Ltd., model DW-2000) (4); Electronic thermometer (Hangzhou Yisida Co. Ltd., measuring range 0-100°C) (5); Electronic balance (Mettler Toledo, Switzerland, model GB204) (6); Full-wavelength microplate reader (USA MD, model SpectraPlus 384) (7); Incubator (Shanghai Jinghong, model DHG-9070A) (8); Micropipettor (9); Rotary slicer (LEICA,RM2235) (10); Pathological tissue drier (Changzhou Haosilin Instrument and Equipment Co. Ltd., tec2500); AD microscope (OLYMPUS,BX43) (11); Water-jacket constant temperature incubator (Shanghai Yuejin Medical Equipment Co. Ltd., PYX-DHS500BS-II) (12); Multifunctional infrared digital thermometer (SHARP, Japan).

### Methods

#### Animal Modeling

(1) Animal modeling by drug castration with GnRH-a injection: GnRH-a (Triptorelin Acetate for Injection) was injected intramuscularly for 7 days to SD adult female rats, and then maintained at 1/5 of the initial dose every day. The dosage of GnRH-a was the routine clinical dosage of 0.05 mg/kg, and the maintenance dosage of 0.01mg/kg, which was obtained after conversion according to the body surface area of humans and rats from the human standard weight to animal non-standard weight:Db = Da.Rab.Sb

(2) Saline injection control group: Normal saline was injected intramuscularly for 7 days to SD adult female rats, and then maintained at 1/5 of the initial dose every day. The purpose of setting up the normal saline control group was to remove the confounding factors in the trial result caused by drug injection.

#### Analysis of Vaginal Smears in Rats

To test whether modeling with the GnRH-a injection was successful, vaginal smears were done once every day two weeks after GnRH-a injection (intramuscular injection at 0.06mg/kg for one week, intramuscular injection at 0.012mg/kg for one week) to observe the modeling effect. The vaginal smear analytical method was used for examination. A dropper filled with 0.9% normal saline was gently inserted into the vagina of the rat by about 1-2cm, and normal saline was injected and then sucked out, which was repeated two to three times. The vaginal irrigation solution in the dropper was dripped on the glass slide. The number of semitransparent flat epidermal cells in the liquid was observed under the inverted height electron microscope for 5 to 7 days. If the number of flat epidermal cells did not increase on the continuous observation days, it showed that GnRH-a modeling was successful; otherwise, it failed, and the rat should be excluded as an experimental subject. The specific method has been described in detail with the same series of tests (14).

#### Drug Formulation Configuration and Gavage

All rats started drug treatment after successful model identification (in the 4th week of GnRH-a injection). The experimental drugs were prepared as follows: estradiol valerate (E2) and black cohosh preparation (ICR) tablets were treated with ultrasound and dissolved in sterile saline to form a uniform turbid solution. The following doses were given by intragastric administration from 8:00 a.m. to 9:00 a.m. every day. GnRH-a + NS group: normal saline gavage, 10 mL/kg; NS + NS group: normal saline gavage, 10 mL/kg; GnRH-a + E2 group: E2 gavage, 0.8 mg/kg; GnRH-a + ICR group: ICR gavage, 60mg/kg (crude drug). The rats were weighed every other day, and the dosage was adjusted according to the changes in body weight. The specific method has been described in detail with the same series of tests (14).

#### Specific Experimental Grouping and Medication Intervention Plan

After the experimental animal modeling was identified as successful, the rats were randomly divided into four groups according to the modeling method and drug intervention scheme, with six rats in each group:

(1) GnRH-a injection + normal saline intervention group (GnRH-a + NS): After the GnRH-a injection modeling was identified as successful, normal saline for injection (10 mL/kg) started to be given intragastrically (10 mL/kg) every day from Week 4 after GnRH-a injection, for a total of 28 days;
(2) Normal saline injection control + normal saline intervention group (NS + NS): After modeling of the NS injection control group was identified as successful, normal saline for injection (10 mL/kg) started to be given intragastrically (10 mL/kg) every day from Week 4 after NS injection, for a total of 28 days;
(3) GnRH-a injection + estradiol intervention group (GnRH-a + E2): After the GnRH-a injection modeling was identified as successful, E2 started to be given intragastrically every day (0.8 mg/kg) from Week 4 after GnRH-a injection, for a total of 28 days;
(4) GnRH-a injection + black cohosh preparation intervention group (GnRH-a + ICR): After the GnRH-a injection modeling was identified as successful, ICR started to be given intragastrically every day (60 mg/kg based on crude drugs) from Week 4 after GnRH-a injection, for a total of 28 days.

#### Collection of Bone Tissue Samples and Determination of Bone Mineral Density (BMD) of the Distal Femur of Rats

After the experimental rats were sacrificed, the right femurs of the rats were taken and the fibrous connective tissues such as muscles and ligaments around the bones were removed and stored at -20°C. A dual-energy X-ray bone density scanner and the attached small animal bone density measurement software (Rat Whole Body V 5.73) were used to detect the bone density of the distal femur of the rat. During measurement, each isolated femur specimen was put in a plexiglass box in order, distilled water (with the water level just over the surfaces of all bone specimens) was injected, and then the bone was scanned with the dual-energy X-ray bone density scanner. In the analysis, the bone density of each bone tissue as a whole was measured first, and then the local one-third area of the distal femur was measured. Each area was measured three times and the average value was taken.

#### Automatic Image Analysis System to Measure Morphometric Parameters of Bone Tissue

The pathological changes of the tibia in the rat model were observed by HE staining. The fixed tissue was taken and replaced with EDTA decalcification solution for decalcification. The decalcification solution was changed once a week and the decalcification time was about 8 weeks. After decalcification was completed, the solution was changed to the fixation solution. After 2 days of fixation, an automatic image analysis system was used to observe the morphometric changes of the upper tibia bone tissue in the rat model.

With the American Osteo Measurexp automatic image analysis system, as well as a frozen image input instrument, a research microscope, and a pathological image analyzer used, the rat’s right tibial proximal metaphysis (cancellous bone at a distance of about 1 mm from the epiphyseal cartilage plate) was measured to get the following four indicators (1): Trabecular bone volume (TBV); (2) Trabecular number (TbN) (1/mm^2^); (3) Trabecular thickness (TbTh) (mm); and (4) Trabecular spacing (TbSp) $\bar{X}\pm s$ (mm).

#### Statistical Analysis

The SPSS13.0 software package was used for statistical analysis. All measurement data were expressed by mean ± standard deviation ( $\bar{X}\pm s$ ), and the mean values of multiple samples in a group design were compared with one-way analysis of variance. If the result is significant, the groups were further compared by using Turkey’s test. The count data was tested by χ2. If the χ2 test results of multiple groups were significant, then a χ2 test was performed on the two groups. *P*<0.05 was considered statistically significant. GraphPad Prism 5 software was used to draw related charts.

## Results

### Comparison of BMD of the Distal Femur of Rats in Each Group

**Figure 1:**
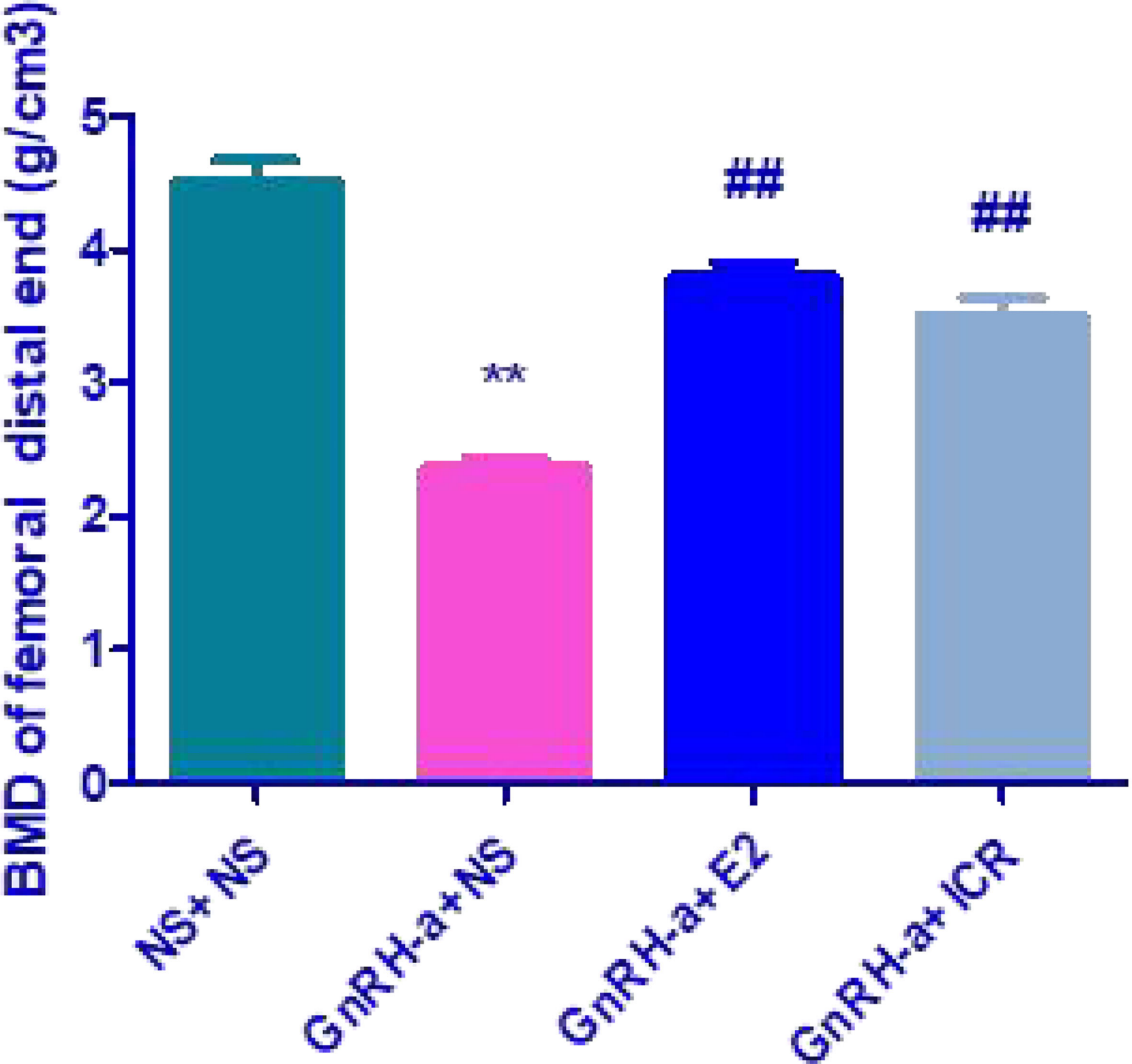
Comparison of the distal femur BMD of rats in each group. **P<0.01 vs. NS + NS; ##*P*<0.01 vs, GnRH-a + NS.

The distal femur BMD of rats in the four groups of NS + NS, GnRH-a + NS, GnRH-a + E2, and GnRH-a + ICR was (4.518 ± 0.3698) g/cm^3^, (2.363 ± 0.1598) g/cm^3^, (3.797 ± 0.2579) g/cm^3^ and (3.511 ± 0.3197) g/cm^3^ (see **Figure 1**). There was a significant difference in BMD between the four groups of (*P*<0.01). The BMD level of the GnRH-a + NS group was significantly lower than that of the NS + NS, GnRH-a + E2, and GnRH-a + ICR groups (*P*<0.01), while that of the GnRH-a + E2 and GnRH-a + ICR groups was slightly lower than that of the NS + NS group, but the difference between the three groups was not statistically significant (*P*>0.05).

### Pathological Changes of Tibia in Each Group

**Figure 2:**
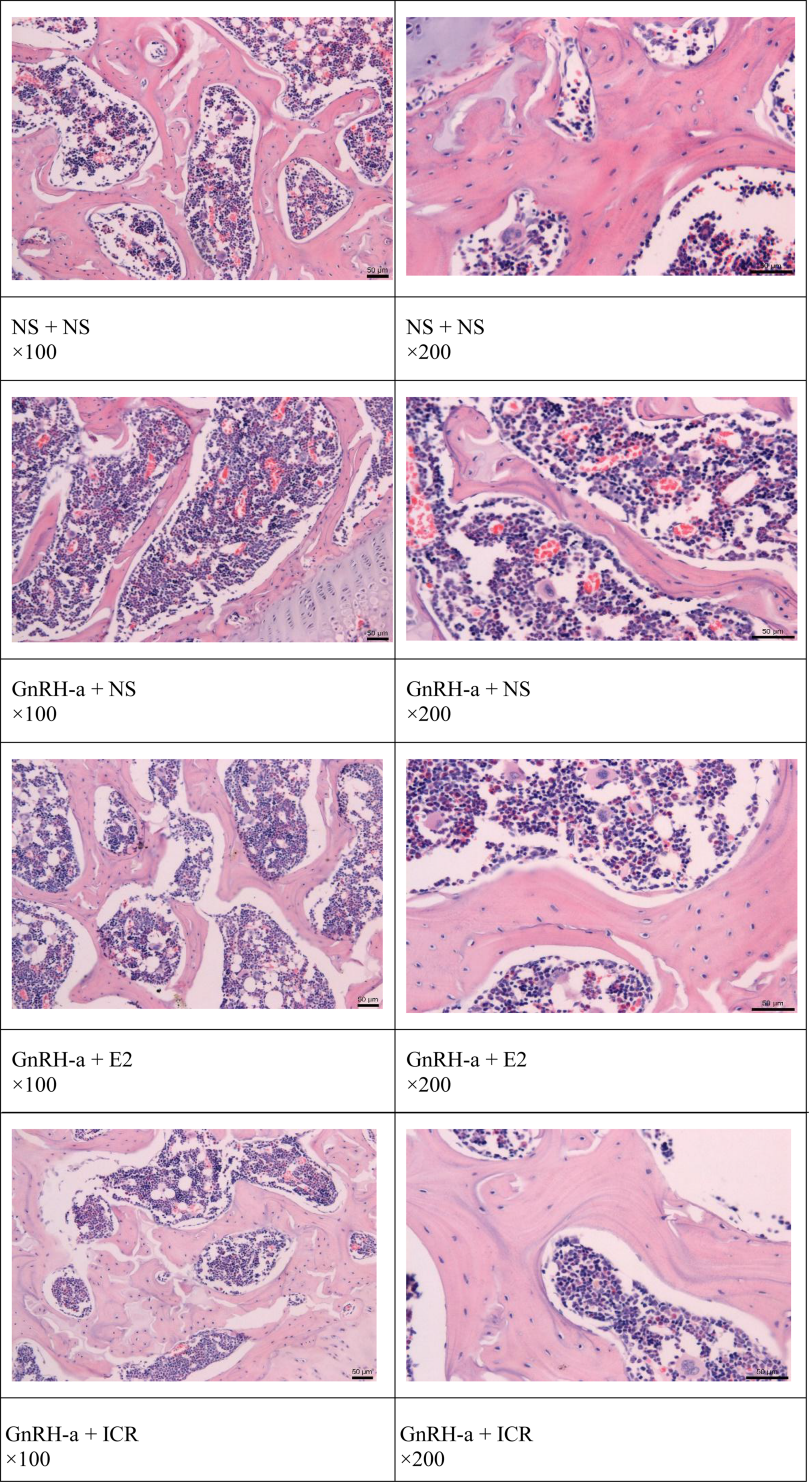
Pathological changes of the tibia in each group.

The morphology and structure of the tibia trabecular bone of the rats in the NS + NS control group were observed under a microscope, with no thinning or fracture, a normal arch structure, no osteoclasts around the trabecular bone, and no bone depression in the bone cells (**Figure 2** NS + NS). Part of the bone trabecula in the GnRH-a + NS group became thinner, and the arched structure disappeared, but the trabecular bone was not broken, and no obvious bone depression was discovered (**Figure 2** GnRH-a + NS). Compared with the NS + NS control group, the morphology and structure of tibial trabeculae in the GnRH-a + E2 and GnRH-a + ICR groups were basically normal, without obvious thinning or fracture, and the arch structure was basically normal (**Figure 2** GnRH-a + E2 and GnRH-a + ICR).

### Comparison of Histomorphometric Parameters of Tibia Bone in Each Group

**Figure 3:**
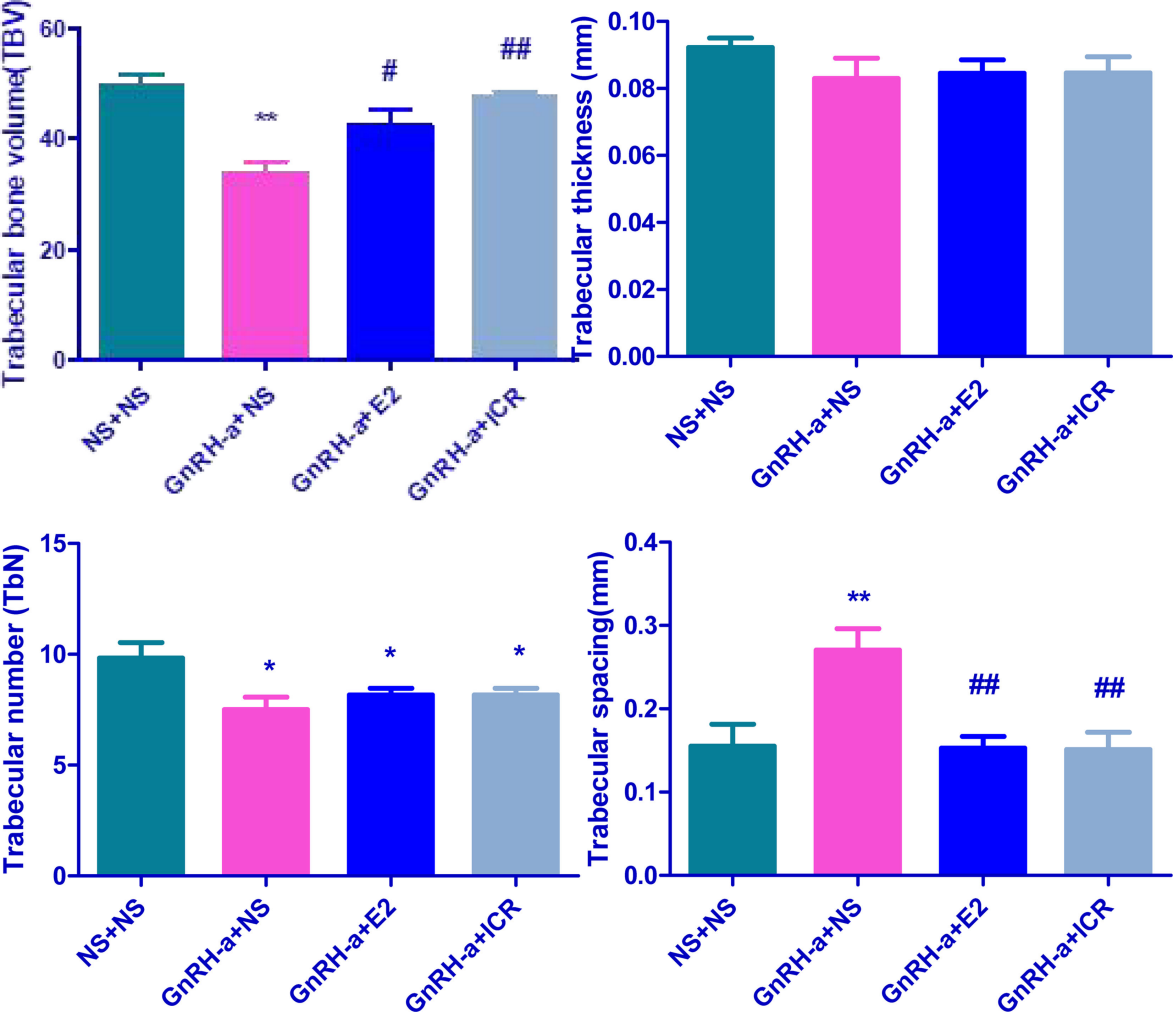
Comparison of histometric parameters of tibia bone in each group. *P < 0.05, **P < 0.01 vs. NS + NS; #*P* < 0.05, ##*P* < 0.01 vs. GnRH-a + NS.

The four groups of rats (NS + NS, GnRH-a + NS, GnRH-a + E2, and GnRH-a + ICR) exhibited significant differences in TBV (*P*<0.01). The TBV in the GnRH-a + NS group was significantly lower than that of the NS + NS, GnRH-a + E2 and GnRH-a + ICR groups (*P*<0.01, *P*<0.05, *P*<0.01). The differences between the NS + NS, GnRH-a + E2 and GnRH-a + ICR groups (*P*>0.05) were not statistically significant (**Figure 3** TBV). The differences in TbTh between the four groups of rats (NS + NS, GnRH-a + NS, GnRH-a + E2, and GnRH-a + ICR) (*P*>0.05) were not statistically significant. GnRH-a + NS, GnRH-a + E2, and GnRH-a + ICR groups showed a downward trend compared with the NS + NS group. Although the values of the GnRH-a + E2 and GnRH-a + ICR groups were slightly higher than that of the GnRH-a + NS group, statistical analysis results showed that the difference was not statistically significant (*P*>0.05) (**Figure 3** TbTh). The four groups of rats (NS + NS, GnRH-a + NS, GnRH-a + E2, and GnRH-a + ICR) had significant differences in TbN (*P*<0.05), and the GnRH-a + NS, GnRH-a + E2, and GnRH-a + ICR groups all showed significant drops compared with the NS + NS group (*P*<0.05). GnRH-a + E2 and GnRH-a + ICR groups showed a slight upward trend compared with the GnRH-a + NS group, but the differences between the three groups were not statistically significant (*P*>0.05) (**Figure 3** TbN). The four groups of rats (NS + NS, GnRH-a + NS, GnRH-a + E2, and GnRH-a + ICR) had significant differences in TbSp (*P*<0.01). The TbSp in the GnRH-a + NS group was significantly higher than that of the NS + NS, GnRH-a + E2, and GnRH-a + ICR groups (*P*<0.01). The differences between the three groups of NS + NS, GnRH-a + E2, and GnRH-a + ICR groups were not statistically significant (*P*>0.05) (**Figure 3** TbSp) (**Table 1**).

**Table 1:** Comparison of histometric parameters of tibia bone in each group of rats.

Group	N	TBV	TbTh (mm)	TbN (1/mm)	TbSp (mm)
NS + NS	6	49.55 ± 5.02	0.09218 ± 0.007080	9.833 ± 1.722	0.1553 ± 0.06430
GnRH-a + NS	6	33.86 ± 5.15**	0.08292 ± 0.01504	7.500 ± 1.378*	0.2704 ± 0.06318**
GnRH-a + E2	6	42.42 ± 7.26^#^	0.08453 ± 0.009828	8.167 ± 0.7528*	0.1528 ± 0.03457^##^
GnRH-a + ICR	6	47.55 ± 2.61^##^	0.08462 ± 0.01192	8.167 ± 0.7528*	0.1512 ± 0.05036^##^

*P < 0.05, **P < 0.01 vs. NS + NS; ^#^P < 0.05, ^##^P < 0.01 vs. GnRH-a + NS.

## Discussion

EMS is prone to relapse after surgery and GnRH-a plays a very important role in postoperative drug treatment (15). The main side effects after GnRH-a treatment are perimenopausal symptoms and osteoporosis caused by a low estrogen level, and the post-treatment symptoms of low estrogen are obvious. Long-term treatment can cause serious bone loss and the effect of GnRH-a treatment on bone metabolism may be long-lasting and far-reaching (4, 16). Therefore, someone advocated using small dose estrogen and progesterone 3 to 6 months after GnRH-a injection, which is the add-back therapy (17–19). By adding estrogen, estradiol can control ectopic lesions without affecting bone metabolism (20).

However, the low-dose estrogen used in the add-back therapy may still increase the estrogen level in the patients. Excessive estrogen levels may lead to the growth of EMS lesions and reoccurrence of pain. Meanwhile, the use of hormones may increase the risk of hormone-dependent tumors (21). Therefore, finding other effective and safe add-back drugs requires an urgent solution.

As a natural botanical medicine, black cohosh has been used in European and American markets for more than two centuries. The research efforts over the years have developed the following hypotheses on the action mechanism of black cohosh (1). Selective estrogen receptor modulator (SERM) (2); Playing a role through the neurotransmitter serotonin 5-hydroxy tryptamine(5-HT) pathway (3); Antioxidation; and (4) Playing a role through inflammation (22). Among them, the first and second mechanisms are closely related to the relief of perimenopausal symptoms. A case-control study on 949 breast cancer patients and 1524 non-breast cancer patients found that the use of black cohosh can significantly reduce the incidence of breast cancer in patients using hormone replacement therapy (HRT), and found that black cohosh has anti-estrogen, anti-proliferation, and antioxidant effects, suggesting that black cohosh can be used in the prevention and treatment of breast cancer (23).

Black cohosh extract can effectively relieve various perimenopausal symptoms such as hot flashes, mental symptoms, and vaginal atrophy (24, 25). Numerous studies have confirmed the clinical efficacy and safety of black cohosh preparations in perimenopausal syndrome (26–30). Some animal experiments and clinical studies have preliminarily discussed the beneficial effect of black cohosh preparations on osteoporosis (31–34). Some scholars have applied black cohosh preparations to the treatment of perimenopausal symptoms caused by GnRH-a in patients with EMS, and satisfactory results have been achieved (35–38). Previously, our team has confirmed that the effect of black cohosh preparations in treating perimenopausal symptoms in patients with EMS treated with GnRH-a is similar to that of tibolone and that black cohosh preparations have no obvious estrogen-like effect or any significant effect on serum sex hormone levels and endometrial thickness (39).

However, only a few reports in animal experiments and clinical studies have studied whether black cohosh preparations can effectively relieve perimenopausal symptoms caused by GnRH-a and effectively prevent or treat bone mass loss, osteoporosis, and even bone pain caused by long-term use of GnRH-a, and more data are needed to confirm the effects (12, 40). This study will explore the effect of black cohosh preparations on bone metabolism of rat models with perimenopausal symptoms caused by GnRH-a injection and preliminarily identify whether black cohosh preparations play a role in bone protection in GnRH-a add-back therapy, which will guide the correct application of black cohosh preparations in clinical practices. The bone loss caused by GnRH-a treatment is particularly prominent in the lumbar vertebrae and femurs (41, 42). Bone histology metrology is a newly developed method for the quantitative study of bone tissue. It transforms the bone tissue morphology shown in two-dimensional images in bone tissue sections into quantitative data so that the changes in bone structure can be identified at the tissue and cellular level. In this study, a rat model of perimenopausal symptoms was established by GnRH-a injection and then the bone mineral density of the distal femur of the rats was detected using a dual-energy X-ray bone mineral density scanner and the attached small animal bone mineral density measurement software. The bone tissue was decalcified and made into slices. The pathological changes of the tibia in each group of rat models were observed by HE staining. The bone histometric parameters of each group of rats were analyzed by the automatic image analysis system. The relative volume of trabecular bone (TBV), trabecular bone thickness (TbTh), trabecular bone number (TbN), and trabecular bone separation (TbSp), among other indicators, were calculated to explore the effects of different drug interventions on bone metabolism in rats. The results showed that GnRH-a injection can decrease the bone mineral density of the distal femur of rats, while the bone mineral density value increased after the intervention of estradiol or black cohosh preparations. In addition, it was observed under the microscope that the GnRH-a injection can lead to changes in the morphological structure of the tibia trabecula of rats, such as trabecular bone thinning and arch structure disappearance. After the intervention of estradiol or black cohosh preparations, the trabecular bone morphology and structure could be partially restored to normal, and the arched structure basically remained normal. In addition, the analysis results of the fully automated image analysis system in this study suggest that GnRH-a injection can lead to a decrease in the relative volume of TBV, the thickness of TbTh, and the TbN, and an increase in TbSp, an indicator of rat osteoclast activity (bone resorption), and the intervention with black cohosh preparations and estradiol can both reverse this trend to a certain extent. These findings suggest that the use of GnRH-a may lead to bone mass loss or even osteoporosis in patients, and black cohosh preparations and estradiol can reverse this bone loss process to a certain extent. The patient’s bone metabolism plays a certain protective role, which is consistent with the results reported in the relevant literature (8, 9, 33).

In recent years, some literature has mentioned that Vilaprisan is used in the treatment of perimenopausal women. Compared with GnRH-a, Vilaprisan may have a better effect with lower side effects (43, 44). However, this kind of drug is not widely used in China, so GnRH-a is still used in this study, which is also one of the shortcomings of this paper. We also look forward to studying the black cohosh effects used in Vilaprisan treatment. Another deficiency of this paper is that the sample size is small and more sample data may be needed to verify the efficacy of this study.

The osteoprotective effect of black cohosh preparations may be related to its role as a selective estrogen receptor modulator (SERM) (45), which can exhibit estrogen-like effects on bone tissue and activate estrogen receptors in bones (46). In addition, some studies found that black cohosh extract may directly promote osteoblast activity and inhibit the synthesis and resorption of osteoclasts, thereby increasing bone production and reducing bone resorption (10). However, the specific adjustment mechanism has not yet been clarified, and further research is still needed.

In conclusion, The GnRH-a injection could achieve the desired effect. GnRH-a injection may lead to bone mass loss in rats. Black cohosh preparations, like estrogen, may have a certain protective effect on the bone mass loss caused by GnRH-a injection.

## Data Availability Statement

The original contributions presented in the study are included in the article/supplementary material. Further inquiries can be directed to the corresponding authors.

## Ethics Statement

The animal study was reviewed and approved by Ethical Committee on Laboratory Animal Welfare.

## Author Contributions

Conceptualization: JC; Methodology, Software: AZ; Validation: JL; Formal analysis: MB; Investigation: HW; Resources: HY; Data Curation: SZ and WZ; Writing - Original Draft: ZQ; Writing - Review and Editing: ZD; Visualization, Supervision: LS; Project administration: JW; Funding acquisition: JC. All authors contributed to the article and approved the submitted version.

## Funding

This study was funded by the maternal and child health research project of Jiangsu Province (F202138), the Scientific Research Support Program for Postdoctoral of Jiangsu Province (2019K064), and the Scientific Research Support Program for “333 Project” of Jiangsu Province (BRA2019161).

## Conflict of Interest

The authors declare that the research was conducted in the absence of any commercial or financial relationships that could be construed as a potential conflict of interest.

## Publisher’s Note

All claims expressed in this article are solely those of the authors and do not necessarily represent those of their affiliated organizations, or those of the publisher, the editors and the reviewers. Any product that may be evaluated in this article, or claim that may be made by its manufacturer, is not guaranteed or endorsed by the publisher.
